# Comparison of the efficacy and safety of thoracic epidural and paravertebral block in postoperative analgesia after thoracic surgery: a meta-analysis of randomized trials

**DOI:** 10.3389/fmed.2026.1747430

**Published:** 2026-02-17

**Authors:** Xinli Qi, Zanwu Li, Longmei Zhou, Jianhua Wang, Xiaodong Zhang

**Affiliations:** 1 Department of Pancreatic Surgery, Weifang People’s Hospital, Weifang, Shandong, China; 2 Department of Critical Care Medicine, Weifang People’s Hospital, Weifang, Shandong, China

**Keywords:** meta-analysis, pain relief, paravertebral block, thoracic epidural analgesia, thoracic surgery

## Abstract

**Objectives:**

Paravertebral block (PVB) and thoracic epidural analgesia (TEA) are commonly used methods for pain relief after open chest surgery. However, due to their different characteristics, there are still controversies regarding the analgesic effect and safety of these two methods after chest surgery. This report represents the latest meta-analysis on this topic.

**Methods:**

We searched PubMed, Embase, and Cochrane Library and identified randomized controlled trials on the use of paravertebral block and thoracic epidural analgesia after thoracic surgery. Two researchers independently screened the identified studies. The efficacy and safety of the two different analgesic methods were compared and analyzed. A meta-analysis was conducted using RevMan 5.4 software. This study has been registered in PROSPERO (CRD420251208232).

**Results:**

Thirty-five trials were included. Compared with paravertebral block (PVB), thoracic epidural analgesia (TEA) provided significantly lower pain scores at 24 h postoperatively (Resting: MD 0.41, *P* = 0.03; Movement: MD 0.40, *P* = 0.03). However, no significant differences were observed at 48 h. PVB was associated with a significantly lower risk of complications, including hypotension (OR 0.13, *P* < 0.00001), postoperative nausea and vomiting (OR 0.38, *P* = 0.0004), and urinary retention (OR 0.23, *P* < 0.0001). Pulmonary complication rates were comparable between groups (OR 0.61, *P* = 0.06).

**Conclusion:**

While TEA demonstrated slightly superior resting and movement pain control at the 24-h, these differences were no longer significant by 48 h. Most notably, PVB was associated with a significantly lower risk of hypotension, postoperative nausea and vomiting, and urinary retention. Overall, PVB is a safer and equally effective alternative to TEA for thoracic surgery.

## 1 Introduction

Severe chest pain after thoracic (including thoracotomy and minimally invasive surgery assisted by thoracoscopy or robot) is widespread. Therefore, effective perioperative pain management is of paramount importance (1). Traditionally, thoracic epidural analgesia (TEA) has been regarded as the “gold standard” for treating severe pain after thoracic surgery (2). Because it can provide extensive and reliable thoracic sensory block and significantly reduce postoperative pain scores and the dosage of opioid drugs. However, TEA still has some limitations: including cyclic instability, bladder dysfunction, and difficulties in implementation in patients with anticoagulation or anatomical abnormalities (3). This limits its wide applicability in certain high-risk patients or in minimally invasive thoracic surgery rapid recovery pathways (4). Thoracic paravertebral block (PVB) usually results in unilateral and multi-segmental thoracic segmental block. Theoretically, it is possible to achieve targeted pain relief and reduce the extensive inhibition of the sympathetic nerve, Therefore, it can reduce the risk of experiencing severe hypotension and other systemic side effects (5, 6).

Earlier meta-analyses have indicated that in terms of controlling acute pain, PVB and TEA can achieve comparable effects, with a significantly lower incidence of circulatory system complications (7). Nevertheless, there is significant heterogeneity among the studies in terms of blocking techniques (single, multi-segment or continuous catheter placement), types/concentrations of local anesthetics, surgical procedures (open chest vs. VATS vs. major incision surgery), evaluation endpoints and follow-up times, which leads to inconsistent conclusions regarding “which block is superior” for different patient subgroups or specific surgical conditions (8, 9).

Based on the above background, this study aims to systematically and meticulously compare the differences in acute analgesic effects, opioid use, perioperative complications, functional recovery indicators, and short-term outcomes between paravertebral nerve block and thoracic epidural block in different surgical types of thoracic surgery. This will provide evidence-based guidance for the selection of surgical analgesic strategies.

## 2 Methods

### 2.1 Protocol and registration

The reporting of this systematic review adheres to the PRISMA 2020 statement (10). The study protocol was registered prospectively on the International Platform of Registered Systematic Review and Meta-analysis Protocols, with full details available upon request.

### 2.2 Eligibility criteria

Studies were included if they met the following criteria: (1) population: adult patients who need open-chest surgery or minimally invasive thoracic surgery (such as lung or heart surgery); (2) intervention: perform paravertebral nerve block anesthesia; (3) comparison: perform Thoracic epidural anesthesia; and (4) design: randomized controlled trial. The primary outcome was VAS scores at different time points. Secondary outcomes was adverse reactions and complications (Including hypotension, nausea and vomiting, urinary retention, and pulmonary complications).

Exclusion criteria: (1) Chest trauma, lumbar epidural block and epidural opioid-only regimens; (2) TEA with local anesthetics and opioid to PVB with local anesthetics alone; (3) Other types of articles, such as reviews, case reports, conference reports and meta-analyses.

### 2.3 Information sources

We searched PubMed, Embase, and the Cochrane Library from the inception to 1 September 2025. We used the following search terms: “paravertebral block,” “paravertebral anesthesia,” “thoracic epidural,” “thoracic epidural anesthesia,” “randomized controlled trials” and their alternative words, and combined them by “AND” and “OR” reference lists of articles in previous system reviews or meta-analyses were also searched.

### 2.4 Study selection

We removed duplicate records from the initial search, screened the titles and abstracts for relevance, and labeled records as included, excluded or uncertain. We reviewed the full text labeled included or uncertain to identify eligibility of studies. In case of disagreement in the above process, a third reviewer was consulted to reach consensus.

### 2.5 Data extraction

Data extraction was performed using a standardized Excel (Microsoft Corporation) and confirmed by a second reviewer. The relevant information extracted from each study were as follows: author, year, study design, clinical setting, study population, number of patients, and outcomes. Any discrepancy was resolved by discussion between the two reviewers.

### 2.6 Quality assessment

The quality evaluation of the included RCTs was evaluated using the methods recommended by the Cochrane systematic review manual for assessing risk of bias. Any disagreements between the investigators were resolved by third investigators reviewing the original study or consulting the corresponding author.

### 2.7 Statistical analysis

We calculated the pooled odds ratio (OR) for dichotomous outcomes and the mean difference (MD) for continuous outcomes, together with 95% confidence intervals (CIs). The random-effects model was chosen for all analyses because of the anticipated clinical heterogeneity. Heterogeneity across studies was assessed by using the Q statistic with *P*-value and I^2^ statistic. We considered an I^2^ value greater than 50% as significant heterogeneity. A two-sided *P*-value less than 0.05 was considered statistically significant. All statistical analyses were performed using RevMan 5.3 (Nordic Cochrane Center).

## 3 Results

### 3.1 Eligible literature search results and study characteristics

**FIGURE 1:**
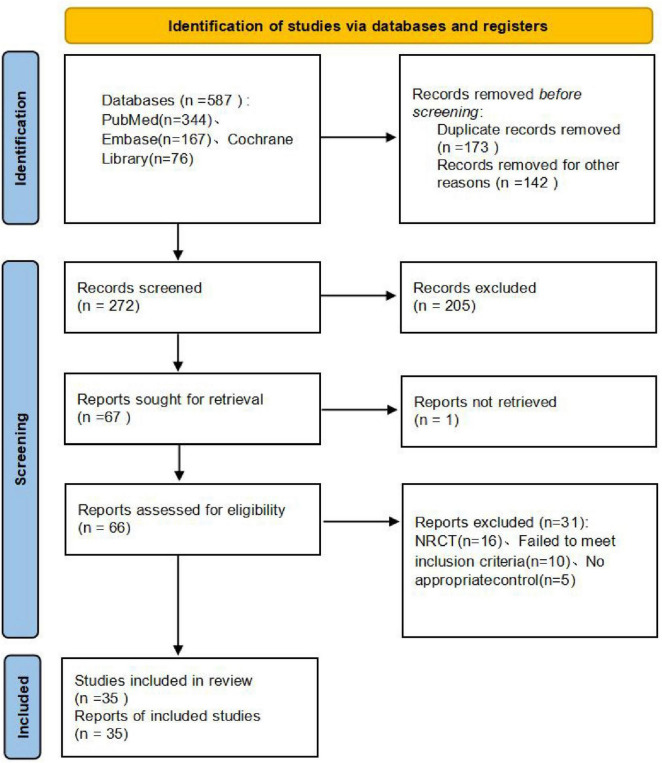
Database search method flow diagram.

We manually searched the references from similar studies. Eventually, 35 RCTs (11–45) involving 2,412 patients were enrolled for this meta-analysis. The flow diagram of the literature search and study selection is shown in Figure 1. The baseline information of the eligible studies is shown in Supplementary Tables 1, 2.

### 3.2 Risk of bias of enrolled trials

**FIGURE 2:**
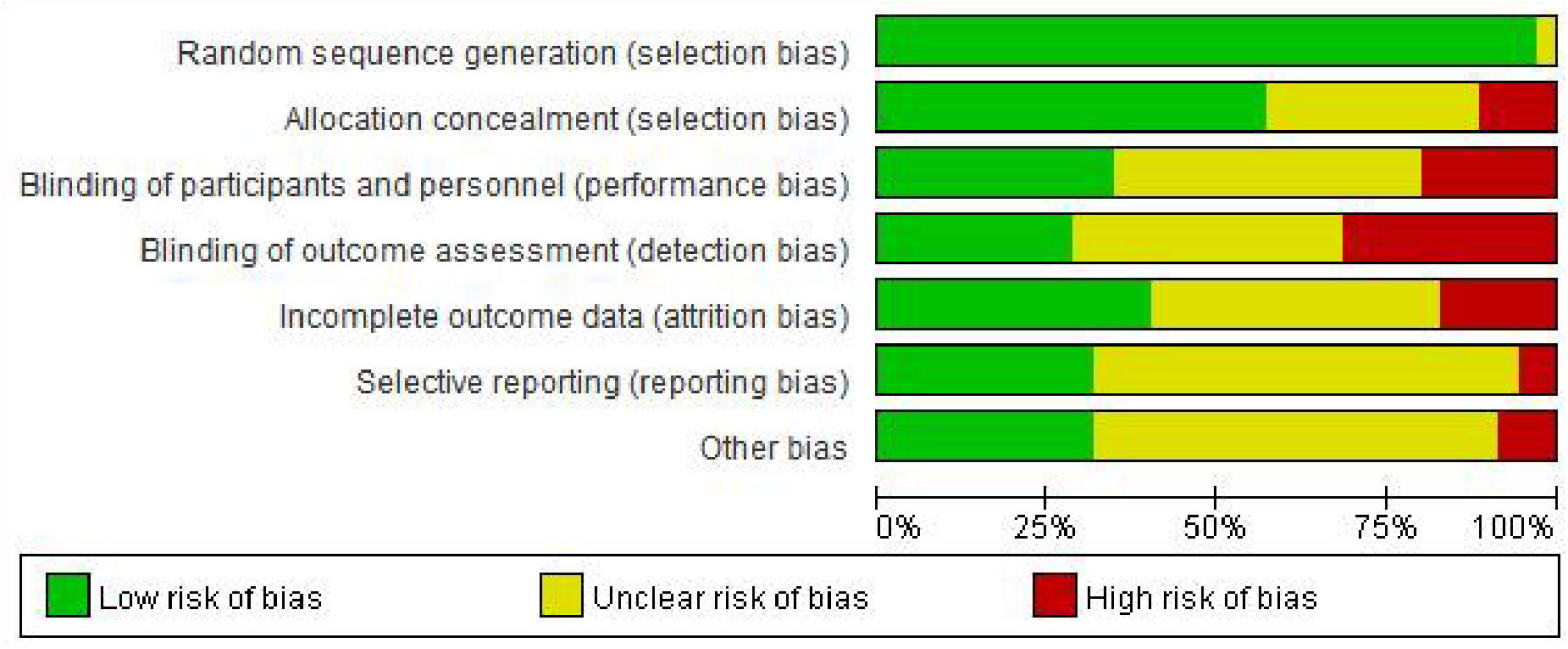
Bias risk of included trials.

The quality evaluation revealed that the overall risk of bias of included trials was deemed to have low or unclear. Due to the intervention nature of the anesthesia method, it is difficult to conduct blind operations for the clinicians and patients. The quality assessment results are shown in Figure 2.

### 3.3 Main outcome

#### 3.3.1 VAS score at rest

**FIGURE 3:**
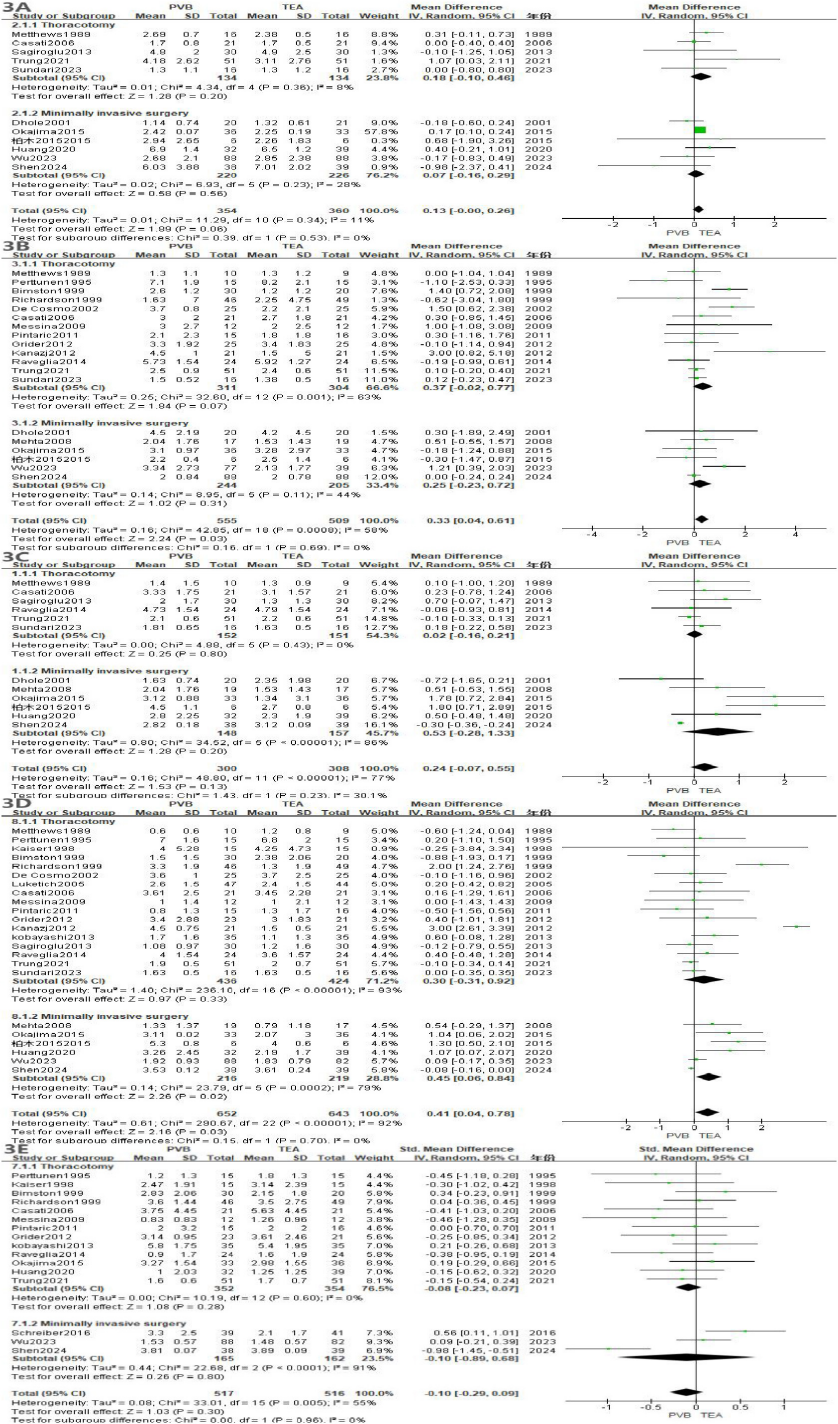
Forest plot of VAS scores in the resting state at different time points for the two groups. **(A)** at 0–4 h, **(B)** at 6–8 h, **(C)** at 12 h, **(D)** at 24 h, **(E)** 48 h.

At rest, meta-analysis of showed that TEA provided significantly better analgesia than PVB at 6–8 h (MD = 0.33, *P* = 0.03, Figure 3) and 24 h (MD = 0.41, *P* = 0.03). No significant differences were observed at 0–4 h (*P* = 0.06), 12 h (*P* = 0.13), or 48 h (*P* = 0.30).

#### 3.3.2 VAS score in the active state

**FIGURE 4:**
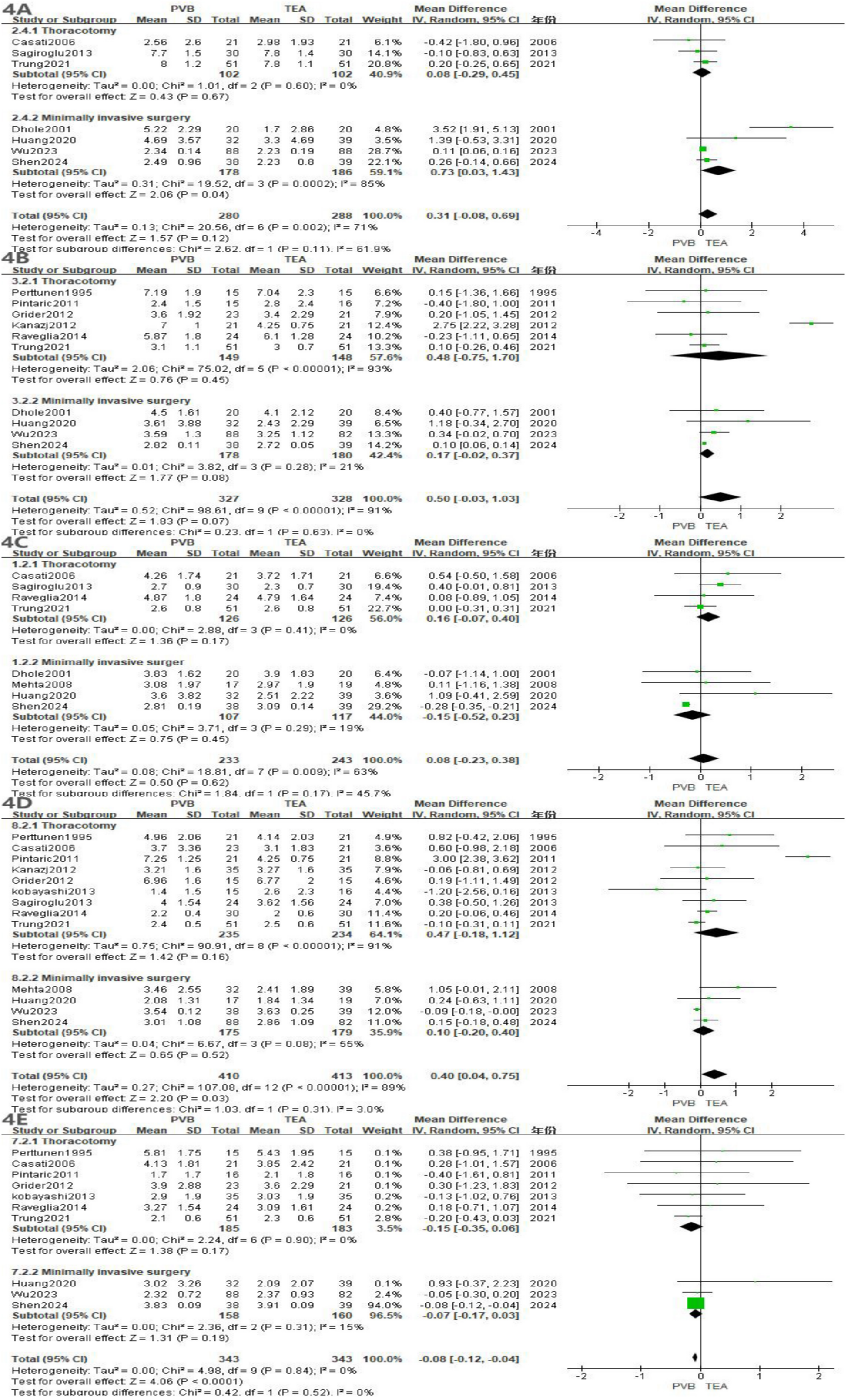
Forest plot of VAS scores in the active state at different time points for the two groups. **(A)** at 0–4 h, **(B)** at 6–8 h, **(C)** at 12 h, **(D)** at 24 h, **(E)** 48 h.

In the active state, meta-analysis of showed that TEA provided better analgesia than PVB at 24 h (MD = 0.40, *P* = 0.03, Figure 4). At 48 h, PVB provided a better analgesic effect (MD = −0.08, *P* < 0.0001). There was no difference in VAS scores between the PVB and TEA groups at 0–4, 6–8, 12 h.

### 3.4 Secondary outcome

#### 3.4.1 Hypotension

**FIGURE 5:**
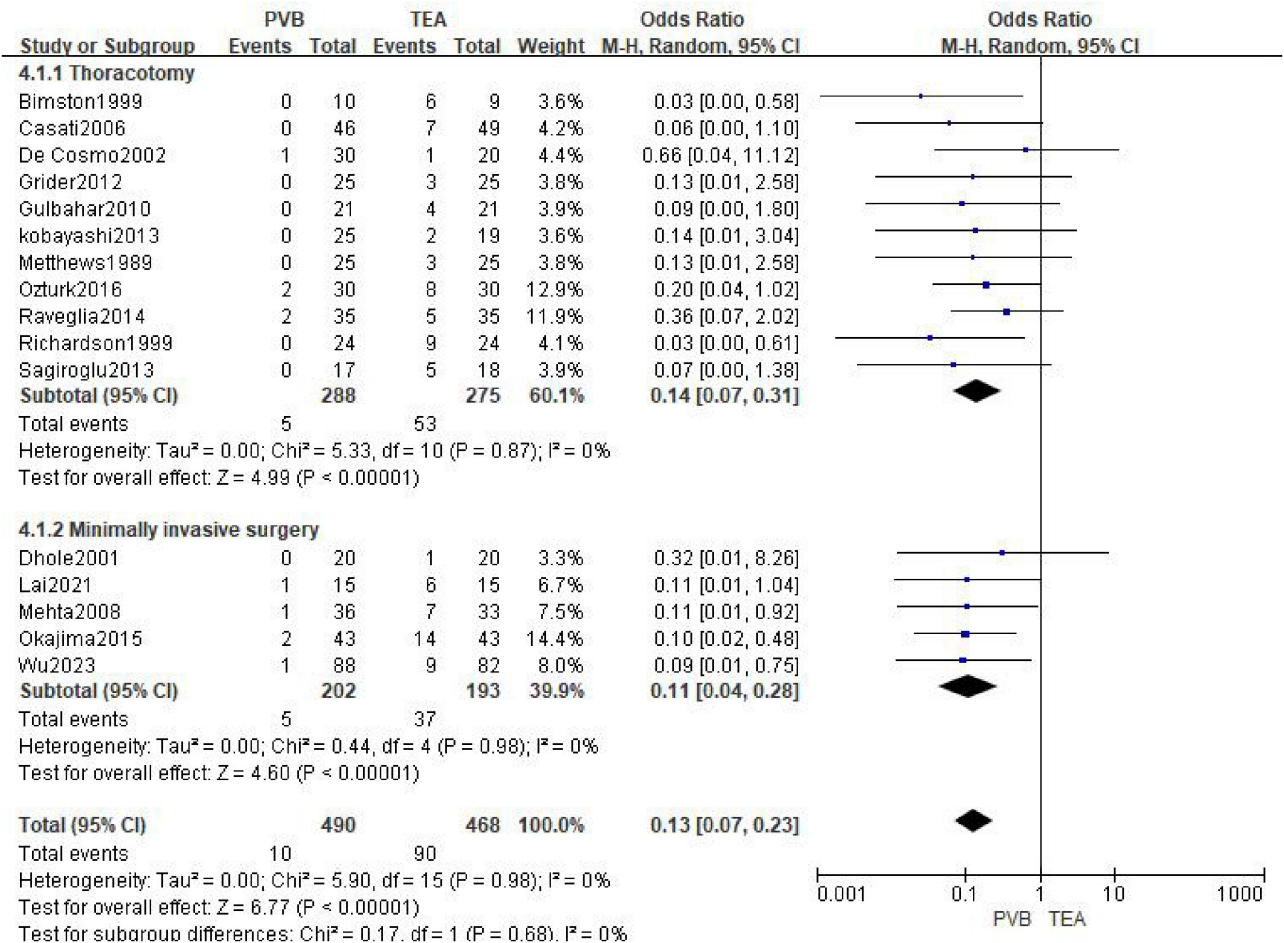
Forest plot showing the occurrence of hypotension in the two groups after surgery.

A total of 16 articles (11–15, 20, 22, 24, 25, 28, 29, 32–34, 43, 44) reported the incidence of postoperative hypotension, involving 490 patients in the PVB group and 468 patients in the TEA group. The meta-analysis using a random-effects model demonstrated that the incidence of hypotension was significantly lower in the PVB group compared with the TEA group (OR = 0.13; 95% CI [0.07, 0.23]; *P* < 0.00001, Figure 5). No statistical heterogeneity was observed across the included studies (I^2^ = 0%; *P* = 0.98).

Subgroup analyses were performed based on the surgical approach (Thoracotomy vs. Minimally Invasive Surgery) to further explore the stability of the results. In the 11 trials involving thoracotomy, PVB was associated with a significantly reduced risk of hypotension compared to TEA (OR = 0.14; 95% CI [0.07, 0.31]; *P* < 0.00001). For patients undergoing Minimally Invasive Surgery (five trials), the PVB group also exhibited a markedly lower incidence of hypotension (OR = 0.11; 95% CI [0.04, 0.28]; *P* < 0.00001).

#### 3.4.2 Nausea and vomiting

**FIGURE 6:**
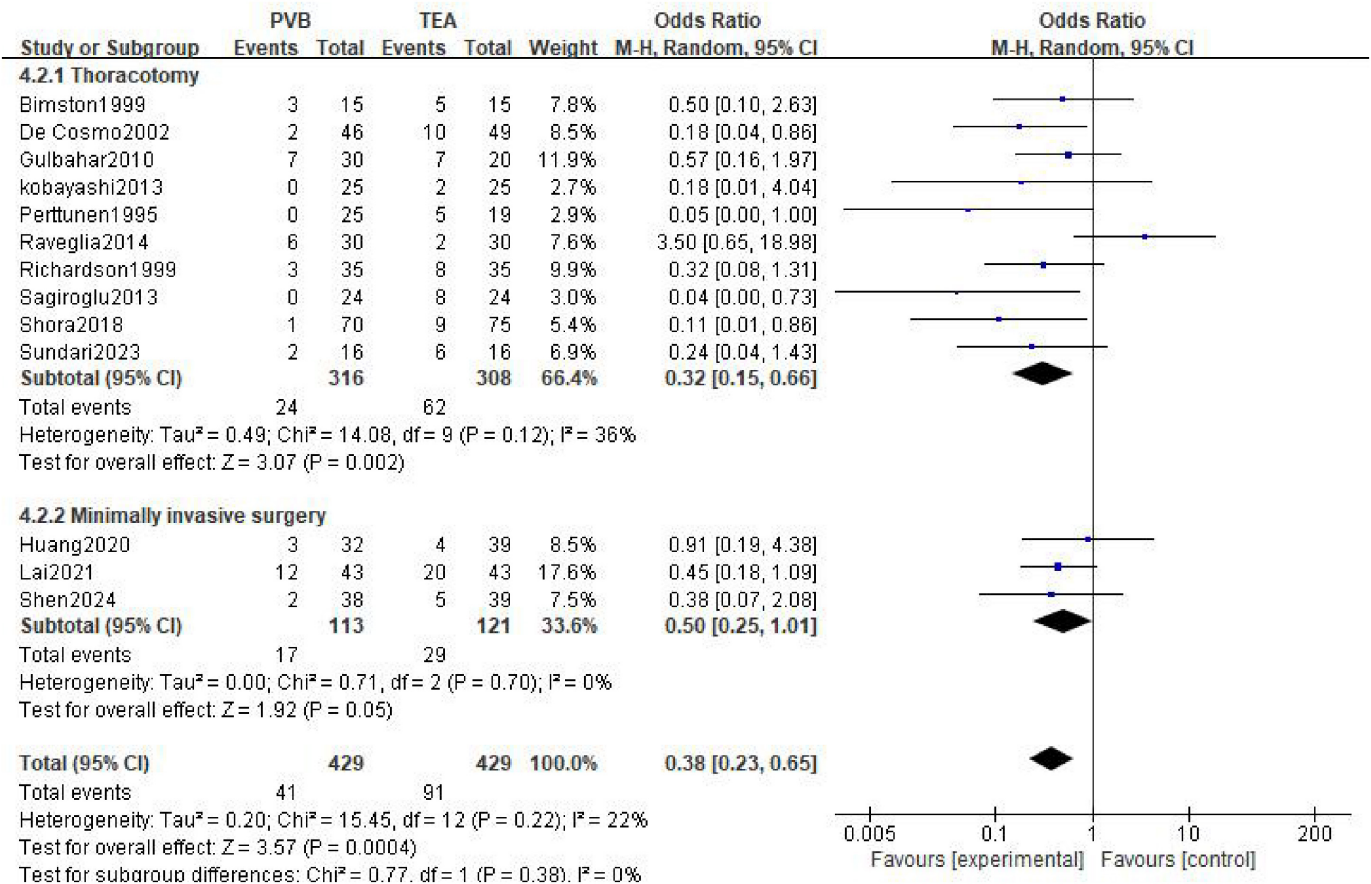
Forest plot showing the occurrence of nausea and vomiting in the two groups after surgery.

A total of 13 studies (11, 15, 16, 20, 22, 31, 33–35, 37, 38, 41, 45) involving 858 patients (429 in the PVB group and 429 in the TEA group) reported the incidence of postoperative nausea and vomiting. The pooled analysis using a random-effects model showed that the PVB group had a significantly lower incidence of nausea and vomiting compared to the TEA group (OR = 0.38; 95% CI [0.23, 0.65]; *P* = 0.0004, Figure 6). Low statistical heterogeneity was observed across the studies (I^2^ = 22%; *P* = 0.22).

In the 10 trials involving thoracotomy, PVB was associated with a significantly reduced risk of nausea and vomiting compared with TEA (OR = 0.32; 95% CI [0.15, 0.66]; *P* = 0.002). For patients undergoing minimally invasive surgery (three trials), the PVB group showed a trend toward a lower incidence of nausea and vomiting, reaching the threshold of statistical significance (OR = 0.50; 95% CI [0.25, 1.01]; *P* = 0.05).

#### 3.4.3 Urinary retention

**FIGURE 7:**
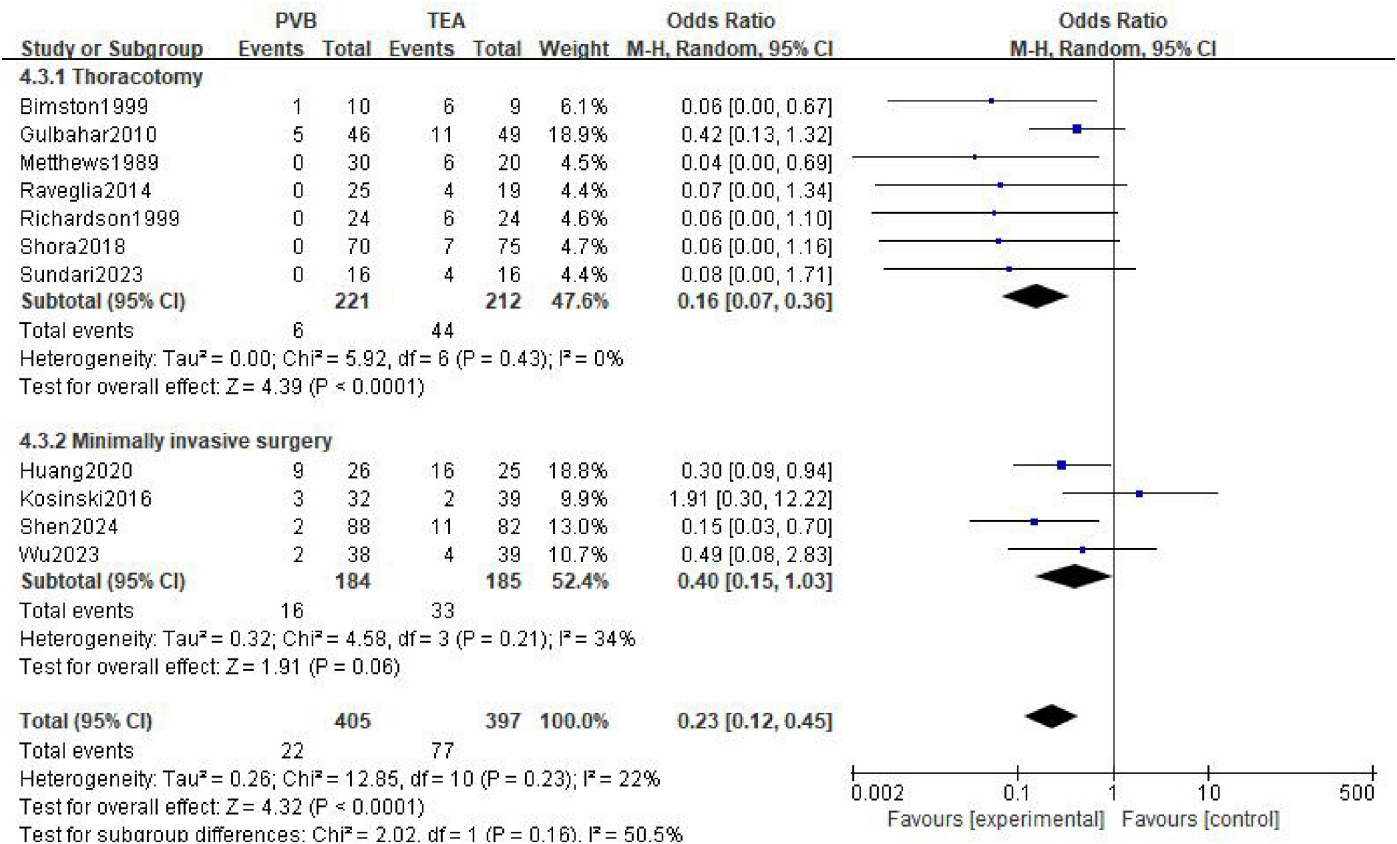
Forest plot showing the occurrence of urinary retention in the two groups after surgery.

A total of 11 studies (11, 15, 16, 21, 24, 32, 33, 36, 37, 40, 43) reported the incidence of postoperative urinary retention, comprising 405 patients in the PVB group and 397 patients in the TEA group. The meta-analysis, utilizing a random-effects model, revealed that the PVB group had a significantly lower incidence of urinary retention compared to the TEA group (OR = 0.23; 95% CI [0.12, 0.45]; *P* < 0.0001, Figure 7). Low statistical heterogeneity was observed across the included trials (I^2^ = 22%; *P* = 0.23).

In the seven trials involving thoracotomy, PVB was associated with a significantly reduced risk of urinary retention compared with TEA (OR = 0.16; 95% CI [0.07, 0.36]; *P* < 0.0001). For patients undergoing minimally invasive surgery (four trials), the PVB group showed a lower incidence of urinary retention (OR = 0.40; 95% CI [0.15, 1.03]), although this result reached the threshold of marginal statistical significance (*P* = 0.06).

#### 3.4.4 Pulmonary complications

**FIGURE 8:**
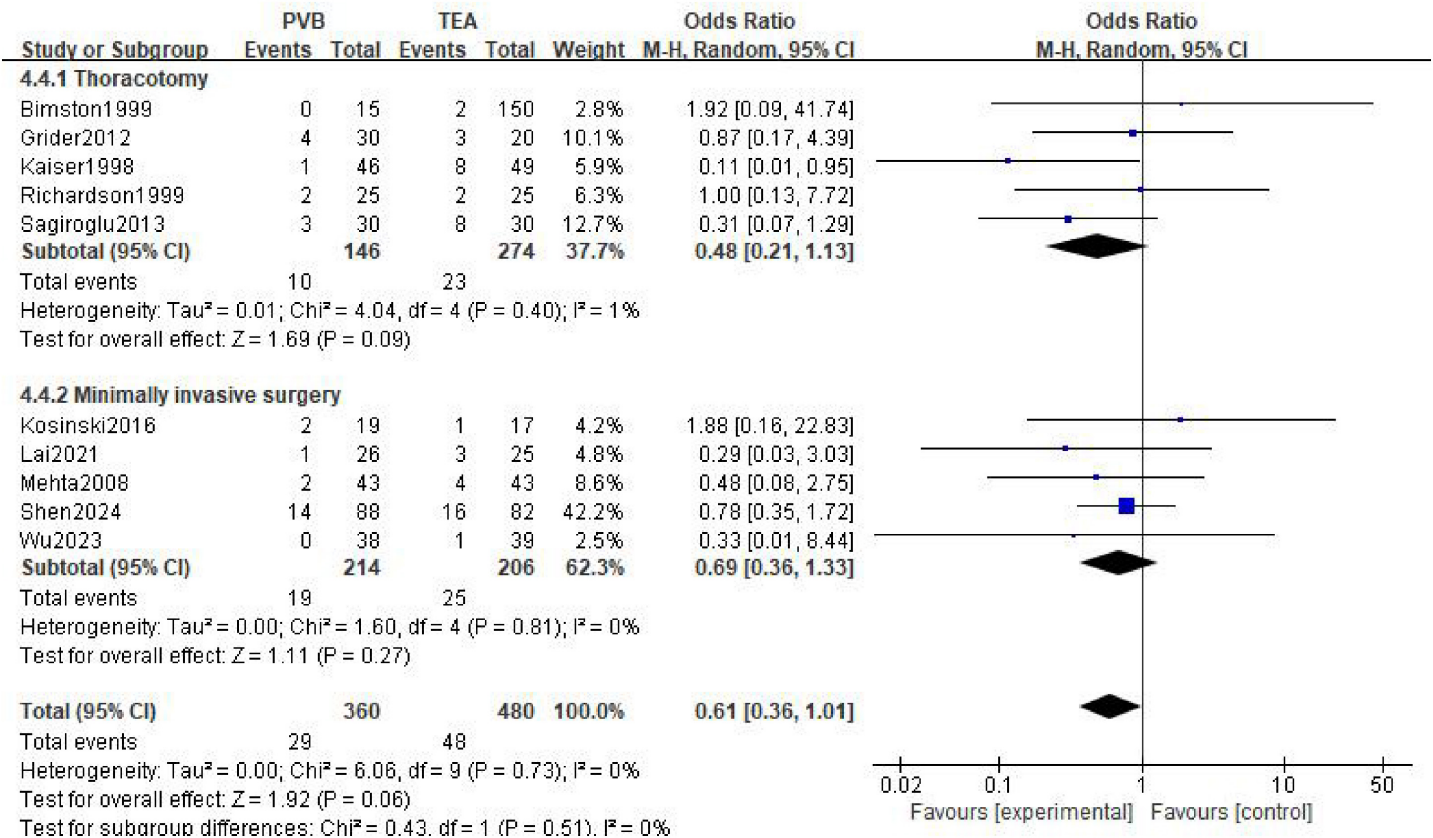
Forest plot showing the occurrence of pulmonary complications in the two groups after surgery.

A total of 10 studies (11, 14, 17, 21, 22, 25, 33, 34, 36, 43) reported the incidence of postoperative pulmonary complications, involving 360 patients in the PVB group and 480 patients in the TEA group. The meta-analysis, performed using a random-effects model, showed that there was no statistically significant difference in the incidence of pulmonary complications between the PVB and TEA groups (OR = 0.61; 95% CI [0.36, 1.01]; *P* = 0.06, Figure 8). No statistical heterogeneity was observed across the included trials (I^2^ = 0%; *P* = 0.73).

Subgroup analyses were conducted to evaluate whether the surgical approach influenced the comparative risk of pulmonary complications. In the five trials involving thoracotomy, the incidence of pulmonary complications did not differ significantly between the two groups (OR = 0.48; 95% CI [0.21, 1.13]; *P* = 0.09). Similarly, for patients undergoing minimally invasive surgery (5 trials), no significant difference was observed between PVB and TEA (OR = 0.69; 95% CI [0.36, 1.33]; *P* = 0.27).

## 4 Discussion

This study compared the analgesic effects and safety of paravertebral block (PVB) and thoracic epidural analgesia (TEA) in thoracic surgery. This meta-analysis included a total of 38 RCT studies, involving 2,928 patients. As far as we know, this is the meta-analysis that includes the largest number of studies to date. The current evidence indicates that in patients undergoing thoracic surgery, the analgesic effect of TEA is superior to that of PVB at 6–8 h after the operation. At 48 h after the operation, the analgesic effect of PVB might be better. At other times, the analgesic effect provided by PVB is comparable to that of TEA, but it is associated with a lower incidence of hypotension, nausea and vomiting, urinary retention and pulmonary complications.

Pain management for patients after thoracic surgery is a common clinical challenge (46). The pain from thoracic surgery includes both the body surface pain caused by incision and traction on the chest wall, as well as the visceral-like pain caused by involvement of the pleura and mediastinum (47). Thoracic epidural anesthesia (TEA) is the most commonly used anesthesia method in thoracic surgery (48). TEA achieves bilateral multi-segmental sympathetic nerve blockage by injecting local anesthetic into the epidural space, thereby achieving extensive thoracic segmental analgesic effects (49). However, extensive sympathetic nerve blockage associated with peripheral blood vessel dilation and hypotension, especially in cases of inadequate intravascular volume, elderly individuals, or those with cardiovascular comorbidities (50). In addition, TEA also carries the risks of nerve damage and paraplegia (51). Sometimes, the epidural technique may also fail due to anatomical reasons. Paravertebral block (PVB) involves injecting local anesthetic into the interspace adjacent to the thoracic vertebrae, which results in a unilateral, multi-segmental but more localized block. PVB not only provides effective pain relief but also avoids extensive sympathetic nerve inhibition (52). Due to the difference in their blocking ranges, TEA may offer better analgesic effects in certain cases of severe or early postoperative pain. While PVB may have an advantage in unilateral surgeries or in patients requiring hemodynamic stability. This is consistent with our research results. In addition, the incidence of urinary retention, nausea and vomiting was lower in the PVB group. This is consistent with the previous research results. Unlike previous studies, we found that the analgesic effect of PVB gradually strengthened over time. At 48 h after thoracic surgery, the VAS score of the PVB group was lower than that of the TEA group. Some studies suggest that PVB can completely block neural signals and thereby eliminate the “central sensitization” stimulus. This indicates that PVB may have a greater advantage in alleviating chronic pain following thoracic surgery (53).

Generally speaking, the pain is more pronounced in the early stage after surgery. Therefore, the extensive bilateral block provided by TEA can more comprehensively suppress these pains. Thoracotomy usually leads to extensive changes in the tension of the chest wall and intercostal muscles, intense irritation of the pleura, and severe postoperative pain. Minimally invasive thoracoscopic surgery (VATS) has smaller incisions, less tissue trauma and less internal organ traction (54). The pain is mainly caused by the single incision on one side and the traction on the chest wall. Therefore, theoretically speaking, the surgical method can affect the efficacy of these two anesthesia methods. However, our meta-analysis confirmed that there was no significant difference in VAS scores between the PVB group and the TEA group except for the 24 h period. This indicates that PVB can provide effective analgesic effects in both open-chest surgeries and minimally invasive surgeries. The efficacy of PVB is highly dependent on the number of blocked segments, the injection volume/concentration, and whether continuous catheterization is used (55). Ultrasound or image-guided multi-segment or continuous administration can significantly enhance the analgesic persistence and uniformity of PVB. Make it comparable to TEA in terms of short-term and even medium-term pain relief. The continuous catheter technology of TEA is mature, but it is limited when there are risks of anticoagulation, spinal deformity or puncture failure. The inconsistencies in some of the comparison results can be explained by the differences in the blocking methods (single vs continuous) and the variations in the drug formulations among the studies (56).

Although the overall complication rate of PVB is relatively low, there are still risks such as pneumothorax, systemic toxicity of local anesthetics, and hematoma. Moreover, caution is still needed in patients with severe coagulation disorders (57). The severe complications of TEA (such as epidural hematoma, widespread hypotension, and infection) carry a higher risk in patients who are anticoagulated or have unstable hemodynamics (56). Therefore, for patients with anticoagulation requirements, cardiovascular dysfunction, or those who expect rapid recovery during or after the surgery and reduced hemodynamic fluctuations, PVB is more attractive; however, in cases of severe chest wall tension, where extensive longitudinal coverage is required or visceral pain is dominant, TEA still offers advantages (31).

There are differences among various studies in aspects such as blocking techniques (single vs. continuous), types/concentrations of local anesthetics, time points for measuring analgesic outcomes, and the inclusion of different types of surgeries (58). As a result, some of the results show significant heterogeneity when combined. Larger sample sizes, stratification (by surgical type and comorbidities), and clearly defined endpoints are required for further confirmation through randomized controlled trials.

In summary, both PVB and TEA have distinct strengths and limitations in perioperative pain management for thoracic surgery. TEA produces a wider and more extensive sensory and sympathetic block, making it particularly suitable for open thoracic procedures that require bilateral or multisegmental coverage, as well as for situations involving pronounced visceral pain. In contrast, PVB mainly achieves unilateral and segmented anesthesia. Due to the relatively lower sympathetic nerve block it causes, PVB may be more suitable for minimally invasive thoracic surgeries with smaller wounds. Moreover, PVB offers better hemodynamic stability and fewer related complications. Therefore, in clinical practice, the choice between these two techniques should be individualized, taking into account surgical type, patient comorbidities, anticoagulant use, and the overall goals of enhanced recovery. Future well-designed comparative studies are needed to further refine the optimal analgesic strategies for different patient subgroups.
