# Supplementary material for: Comparison of the efficacy and safety of thoracic epidural and paravertebral block in postoperative analgesia after thoracic surgery: a meta-analysis of randomized trials
Source: Front Med (Lausanne). 2026 Feb 17;13:1747430. doi: 10.3389/fmed.2026.1747430 (PMC12953078; doi:10.3389/fmed.2026.1747430)
Supplement: Supplementary file 1 [file Table_1.docx]

| Published  year | First author | Country | Surgical  approach | PVB | | | TEA | | | Ultrasound | Additional  analgesics |
| --- | --- | --- | --- | --- | --- | --- | --- | --- | --- | --- | --- |
|  |  |  |  | Amount | Location | Anesthetics dose(mg) | Amount | Location | Anesthetics dose(mg) |  |  |
| 1999 | Bimston | America | Thoracotomy | 25 | - | 0.1% bupivacaine + 10 µg/mL fentanyl (loading dose: 18 mL 0.5% bupivacaine + 2 mL fentanyl) | 25 | - | 0.1% bupivacaine + 10 µg/mL fentanyl (loading dose: 18 mL 0.5% bupivacaine + 2 mL fentanyl) | NO | - |
| 2006 | Casati | Italy | Thoracotomy | 21 | T4-T6 | 0.75% ropivacaine 15 mL (injected in three points) + 0.2% ropivacaine continuous infusion at 5-10 mL/h | 21 | T5-T6/T6-T7 | 0.75% ropivacaine 5 mL (initial dose) + 0.2% ropivacaine continuous infusion at 5-10 mL/h | NO | 5mg intravenous morphine + 1g acetaminophen every 8 hours |
| 2001 | Dhole | India | Minimally invasive  direct coronary  artery bypass | 21 | T4-T5 | 0.5% bupivacaine 8 mL as a loading dose (40 mg) + 0.25% bupivacaine 6 mL/h continuous infusion | 20 | T4-T5 | 0.5% bupivacaine 8 mL as a loading dose (40 mg) + 0.25% bupivacaine 6 mL/h continuous infusion | NO | Ketorolac tromethamine 30mg intramuscular injection (for pain relief when VAS > 5) |
| 2012 | Grider | America | Thoracotomy  (cardiac) | 25 | - | 0.25% bupivacaine, 8 mL/h, continuous infusion | 25 | T6 | EB + O group: 0.25% bupivacaine + hydrocodone 0.01mg/mL (base infusion at 2 mL/h + PCA);  EB group: 0.25% bupivacaine (base infusion at 2 mL/h + PCA) | NO | - |
| 2010 | Gulbahar | Turkey | Thoracotomy | 25 | - | 0.25% bupivacaine (administered at a continuous rate of 0.10 mL/kg/h) | 19 | T7-T10 | 0.25% bupivacaine (administered at a continuous rate of 0.10 mL/kg/h) | NO | - |
| 2020 | Huang | China | Thoracotomy | 77 | T4-T5 | 0.2% Ropivacaine (loading dose: 0.5 mg/kg, background dose: 0.25 mg/kg/h, PC`A: 0.25 mg/kg) | 39 | - | 0.1% Ropivacaine (loading dose: 5 mL, background dose: 5 mL/h) | YES | Flurbiprofen acetate + On-demand use of opioid drugs |
| 2018 | Hutchins | America | pancreatic | 26 | T8 | 0.2% Ropivacaine (7 mL/h per side; for patients weighing less than 60 kg, 6 mL/h) | 27 | T7-T8 | 0.125% bupivacaine + hydrocodone 6 µg/mL (10 mL/h) | YES | - |
| 1998 | Kaiser | Switzerland | Thoracotomy | 15 | T3-T6 | 0.5% bupivacaine (0.1 mL·kg⁻¹·h⁻¹ continuous infusion) | 15 | T5-T6 | 0.25 - 0.375% bupivacaine + fentanyl 2 µg/mL (4 - 8 mL/h) | NO | Nicomorphine (subcutaneous injection, maximum dose of 0.1 mg/kg every 4-6 hours as needed) |
| 2012 | Kanazj | Lebanon | Thoracotomy | 21 | - | 0.25% bupivacaine (loading dose of 20 mL + continuous infusion at 8 mL/h) | 21 | T5-T7 | 0.125% bupivacaine (loading dose of 10 mL + continuous infusion at 8 mL/h) | NO | Acetaminophen (intravenous injection of 1g every 6 hours), Ondansetron (intravenous injection as needed at 4mg) |
| 2013 | Kobayashi | Japan | Thoracotomy | 35 | T4-T7 | 0.375% ropivacaine 10 mL (initial dose) + 0.2% ropivacaine + fentanyl 800 µg (continuous infusion at 5 mL/h) | 35 | T4-T7 | 0.2% ropivacaine 5 mL (initial dose) + 0.2% ropivacaine + fentanyl 800 µg (continuous infusion at 5 mL/h) | NO | - |
| 2016 | Kosinski | Poland | video-assisted thoracoscopic | 26 | - | 0.25% bupivacaine + epinephrine 1:200,000 (loading dose 20 mL, maintenance infusion 0.08 - 0.1 mL/kg/h) | 25 | - | 0.25% bupivacaine + epinephrine 1:200,000 (loading dose 6 mL, maintenance infusion 0.06 - 0.08 mL/kg/h) | NO | 100mg of ketoprofen by vein every 12 hours + 1000mg of acetaminophen by vein every 8 hours + 10mg of morphine by vein. |
| 2021 | Lai | China | Video-  Assisted Thoracoscopic | 43 |  | 0.5% ropivacaine 0.1 mL/kg (initial dose) + 0.5% ropivacaine 0.1 mL/kg/h continuous infusion | 43 | T7-T8 | 0.15% ropivacaine + 6 µg/mL hydrocodone (continuous infusion at 2 mL/h) | NO | Parecoxib 40mg + Intravenous Oxycodone |
| 2005 | Luketich | America | Thoracotomy |  | - | - | 61 | T3-T6 | 0.125% bupivacaine + morphine 0.05 mg/mL (4-8 mL/h) | NO | Morphine PCA (1mg every 8 minutes, maximum 30mg within 4 hours) |
| 2009 | Messina | Italy | Thoracotomy | 12 | T6-T7 | 0.25% levobupivacaine  þfentanyl 1.6 µg/mL  (0.1mL/kg/h) | 12 | T6-T7 | 0.125% levobupivacaine  þfentanyl 2 µg/mL  (0.08mL/kg/h) | NO | Morphine |
| 2008 | Mehta | India | CABG | 17 | T4-T5 | 0.5% bupivacaine 8 mL as a loading dose (40 mg) + 0.25% bupivacaine at a rate of 0.1 mL/kg/h for continuous infusion | 19 | C7-T1 | 0.5% bupivacaine 8 mL as a loading dose (40 mg) + 0.25% bupivacaine at a rate of 0.1 mL/kg/h for continuous infusion | NO | Diclofenac sodium 75mg, intramuscular injection (when VAS > 5) |
| 1989 | Metthews | Britain | Thoracotomy | 10 | T4-T5 | 0.25% bupivacaine 10 mL (initial dose) + 0.25% bupivacaine 3-10 mL/h continuous infusion | 9 | T4-T5 | 0.25% bupivacaine 10 mL (initial dose) + 0.25% bupivacaine 3-10 mL/h continuous infusion | NO | - |
| 2010 | Mukherjee | India | Thoracotomy | 30 | T5-T6 | 0.25% bupivacaine 15 mL + fentanyl 50 µg (single injection) | 30 | T5-T6 | 0.25% bupivacaine 7.5 mL + fentanyl 50 µg (single injection) | NO |  |
| 2015 | Okajima | Japan | Video-  Assisted Thoracoscopic | 36 | T2/T4/T6/T8 | 0.5% ropivacaine 30 mL (initial) + 0.1% ropivacaine + fentanyl 0.4 mg/day (continuous infusion at 6 mL/h) | 33 | T4 | 0.25-0.375% Ropivacaine 5 - 7 mL (initial) + 0.1% Ropivacaine + Fentanyl 0.4 mg/day (continuous infusion at 2 - 4 mL/h) | YES | Lisosofen sodium 60mg orally + Flurbiprofen 50mg intravenously / Diclofenac sodium suppository 50mg + Pethidine 15mg intramuscular injection |
| 2016 | Ozturk | Türkiye | Thoracotomy | 20 | T5-T7 | 0.1% levobupivacaine + morphine 0.1 mg/mL (total volume 100.0 ± 10.4 mL) | 20 | T5-T7 | 0.1% levobupivacaine + morphine 0.1 mg/mL (total volume 104.0 ± 9.9 mL) | YES | Tramadol PCA (10 mg/mL, 20 mg bolus dose, 15-minute lockout time) |
| 1995 | Perttunen | Finland | Thoracotomy | 8 | T3 | 0.25% bupivacaine (loading dose of 8-12 mL, continuous infusion at 4-8 mL/h) | 10 | T5-T7 | 0.25% bupivacaine (loading dose of 8-12 mL, continuous infusion at 4-8 mL/h) | NO | - |
| 2011 | Pintaric | Slovenia | Thoracotomy | 15 | T6-T7 | 0.5% levobupivacaine + 30 µg/kg morphine (dose calculated based on height) | 16 | T6-T7 | 0.25% levobupivacaine + 30 µg/kg morphine (dose calculated based on height) | NO | Intravenous injection of diclofenac sodium 75mg + ofenatadine 30mg every 12 hours + intravenous piroxicam 3mg (when VAS > 4) |
| 2014 | Raveglia | Italy | Thoracotomy | 24 | T5-T7 | 0.3% Ropivacaine (5-10 mL, 10 mg/mL, dissolved in 100 mL of normal saline) | 24 | T5-T7 | 0.001% Fentanyl (10 µg/mL) + 0.1% Bupivacaine | NO | Acetaminophen 500mg taken 4 times a day + Tramadol 50mg (up to 2 times a day) when VAS score is > 6 |
| 1999 | Richardson | Britain | Thoracotomy | 46 | T6-T8 | 0.5% bupivacaine 20 mL (initial) + 0.5% bupivacaine 0.1 mL/kg/h continuous infusion | 49 | T7-T10 | 0.25% bupivacaine 10-15 mL (initial) + 0.25% bupivacaine 0.1 mL/kg/h continuous infusion | NO | Preoperative morphine 10mg + diclofenac 50mg + diclofenac 50mg every 8 hours after surgery |
| 2013 | Sagiroglu | Turkey | Thoracotomy | 30 | T6-T8 | 0.25% bupivacaine 10 mL (initial) + 0.25% bupivacaine 0.1 mL/kg/h continuous infusion | 30 | T5-T7 | 0.25% bupivacaine 10 mL (initial) + 0.25% bupivacaine 0.1 mL/kg/h continuous infusion | NO | Intravenous acetaminophen 1000mg + diclofenac 50mg every 8 hours + if VAS > 4, intravenous morphine 2mg |
| 2016 | Schreiber | America | Thoracotomy | 39 | T7/T8 | 0.5% ropivacaine 15 mL/each side (initial dose) + 0.2% ropivacaine 7 mL/h/each side for continuous infusion | 41 | T7-T9 | 0.2% ropivacaine, 5-6 mL/h, continuous infusion | NO | - |
| 2024 | Shen | China | Video-  Assisted Thoracoscopic | 40 | T4-5 | 0.375% Ropivacaine 20 mL (75 mg) | 40 | T5 | 0.375% ropivacaine (3 mL test dose + 4-5 mL/h continuous infusion) + 2 mg morphine as a loading dose | YES | Sufentanil PCIA (background dose of 1mg/h, single dose of 2mL, lock-in time of 15 minutes) + Ketorolac tromethamine 30mg intramuscular injection (rescue analgesia) |
| 2018 | Shora | Egypt | Thoracotomy | 70 | T6-T7 | 0.125% bupivacaine + fentanyl 1 µg/mL (6 mL/hour loading dose + 6 mL/hour continuous infusion) | 75 | T6-T7 | 0.125% bupivacaine + fentanyl 1 µg/mL (12 mL loading dose + 12 mL/h continuous infusion) | Yes | When VAS is ≥ 6, administer 2mg of morphine. |
| 2016 | Singh | India | Thoracotomy | 25 | T5-T7 | 0.125% bupivacaine 10 mL as a loading dose + 0.25% bupivacaine 0.1 mL/kg/h continuous infusion (for 24 hours) | 25 | T5-T7 | 0.125% bupivacaine 10 mL as a loading dose + 0.25% bupivacaine 0.1 mL/kg/h continuous infusion (for 24 hours) | NO | Tramadol 50mg (for rescue analgesia when VAS > 3) |
| 2019 | Sondekoppam | Canada | Cesarean section | 35 | T7-T9 | 0.5% Ropivacaine 15 mL (single dose) + 0.2% Ropivacaine 7 mL/h/each side for continuous infusion | 35 | T7-T9 | 0.25% bupivacaine 5 mL (initial) + 0.1% bupivacaine + hydrocodone 10 µg/mL (continuous infusion at 6 mL/h) | YES | Hydromorphone IV-PCA (0.2mg injection, 6-minute lockout time) + 975mg acetaminophen before surgery + 600mg gabapentin + 500mg naproxen |
| 2025 | Spaans | Netherlands | Video-  Assisted Thoracoscopic | 134 | T4-T5 | - | 131 | - | - | NO |  |
| 2023 | Sundari | India | Thoracotomy | 32 | T3-T8 | 0.25% ropivacaine + 50 µg fentanyl (intermittent administration, once every 6 hours) |  | T5-T6 | 0.25% ropivacaine + 50 µg fentanyl (intermittent administration, once every 6 hours) | NO | Fentanyl + Acetaminophen 1g (twice a day) |
| 2017 | Tamura | Japan | Thoracotomy | 36 | T4-T7 | Bupivacaine 0.125_+_  fentanyl 1µ/mL for 48h | 75 | T4-T7 | 0.375% ropivacaine 5mL  þ(300mL) 0.2%  ropivacaine 5mL/h | No | Indomethacin;  indomethacin;  pentazocine;  loxoprofen; celecoxib |
| 2021 | Trung | Vietnam | Thoracotomy | 51 | T4-T8 | 0.125% bupivacaine + 2 µg/mL fentanyl (loading dose + continuous infusion at 0.1 mL/kg/h) | 51 | - | 0.125% bupivacaine + 2 µg/mL fentanyl (loading dose + continuous infusion at 0.1 mL/kg/h) | YES | Morphine PCA (1mg injection, 15-minute lockout time, maximum 10mg within 4 hours) |
| 2023 | Wu | China | Video-  Assisted Thoracoscopic | 88 | T7-T8 | 2.0% Ropivacaine (background infusion at 5 mL/h + 1 mL bolus injection, with a 15-minute lockout period) | 88 | T7-T8 | 2.0% Ropivacaine (background infusion at 5 mL/h + 1 mL bolus injection, with a 15-minute lockout period) | NO | Dizocine 10mg intramuscular injection (for rescue analgesia when VAS > 4) |
| 2020 | kingma | Netherlands | Video-  Assisted Thoracoscopic | 48 | T4-T5 | 0.125% bupivacaine (loading dose of 20 mL, continuous infusion at 8-12 mL/h) | 48 | T5-T8 | 0.25% bupivacaine 5-10 mL (initial) + 0.125% bupivacaine + sufentanil 0.5 mcg/mL (6-14 mL/h) | NO | - |
| 2015 | 柏木 | Japan | Video-  Assisted Thoracoscopic | 6 | T5-T6 | 0.5% ropivacaine 20 mL (initial dose) + 0.2% ropivacaine 6 mL/h continuous infusion for 48 hours | 6 | T5-T6 | 0.375% ropivacaine 10 mL (initial) + 5 mL per hour during the operation + 0.2% ropivacaine 6 mL/h continuously infused for 48 hours after the operation | YES | Use 50mg of flurbiprofen acetate or 50mg of diclofenac when experiencing pain. |
| 2009 | Mohta | India | - | 15 | - | 0.5% bupivacaine 0.3 mL/kg (initial dose) + 0.25% bupivacaine 0.1 - 0.2 mL/kg/h continuous infusion | 15 | - | 0.5% bupivacaine 1-1.5 mL per segment (initial) + 0.125% bupivacaine 0.1-0.2 mL/kg/h continuous infusion | NO | - |
